# A Website Supporting Sensitive Religious and Cultural Advance Care Planning (ACPTalk): Formative and Summative Evaluation

**DOI:** 10.2196/resprot.8572

**Published:** 2018-04-16

**Authors:** Amanda Pereira-Salgado, Patrick Mader, Clare O'Callaghan, Leanne Boyd

**Affiliations:** ^1^ Centre for Nursing Research Cabrini Institute Malvern, VIC Australia; ^2^ Faculty of Medicine, Nursing and Health Sciences Monash University Clayton, VIC Australia; ^3^ Palliative and Supportive Care Research Department Cabrini Institute Malvern, VIC Australia; ^4^ Departments of Psychosocial Cancer Care and Medicine St Vincent's Hospital, The University of Melbourne Fitzroy, VIC Australia; ^5^ Institute for Ethics and Society The University of Notre Dame Sydney, NSW Australia; ^6^ Cabrini Institute Malvern, VIC Australia; ^7^ Australian Catholic University Fitzroy, VIC Australia

**Keywords:** advance care planning, ehealth, religion, culture, health personnel

## Abstract

**Background:**

Advance care planning (ACP) promotes conversations about future health care needs, enacted if a person is incapable of making decisions at end-of-life that may be communicated through written documentation such as advance care directives. To meet the needs of multicultural and multifaith populations in Australia, an advance care planning website, ACP*Talk,* was funded to support health professionals in conducting conversations within diverse religious and cultural populations. ACP*Talk* aimed to provide religion-specific advance care planning content and complement existing resources.

**Objective:**

The purpose of this paper was to utilize the context, input, process, and product (CIPP) framework to conduct a formative and summative evaluation of ACP*Talk*.

**Methods:**

The CIPP framework was used, which revolves around 4 aspects of evaluation: context, input, process, and product. Context: health professionals’ solutions for the website were determined through thematic analysis of exploratory key stakeholder interviews. Included religions were determined through an environmental scan, Australian population statistics, and documentary analysis of project steering committee meeting minutes.

Input: Project implementation and challenges were examined through documentary analysis of project protocols and meeting minutes.

Process: To ensure religion-specific content was accurate and appropriate, a website prototype was built with content review and functionality testing by representatives from religious and cultural organizations and other interested health care organizations who completed a Web-based survey.

Product: Website analytics were used to report utilization, and stakeholder perceptions were captured through interviews and a website survey.

**Results:**

Context: A total of 16 key stakeholder health professional (7 general practitioners, 2 primary health nurses, and 7 palliative care nurses) interviews were analyzed. Website solutions included religious and cultural information, communication ideas, legal information, downloadable content, and Web-based accessibility. Christian and non-Christian faiths were to be included in the religion-specific content.

Input: Difficulties gaining consensus on religion-specific content were overcome by further state and national religious organizations providing feedback.

Process: A total of 37 content reviewers included representatives of religious and cultural organizations (n=29), health care (n=5), and community organizations (n=3). The majority strongly agree or agree that the content used appropriate language and tone (92%, 34/37), would support health professionals (89%, 33/37), and was accurate (83%, 24/29).

Product: Resource usage within the first 9 months was 12,957 page views in 4260 sessions; majority were (83.45%, 3555/4260) from Australia. A total of 107 Australian-based users completed the website survey; most felt information was accurate (77.6%, 83/107), easy to understand (82.2%, 88/107), useful (86.0%, 92/107), and appropriate (86.0%, 92/107). A total of 20 nurses (general practice n=10, palliative care n=8, and both disciplines n=2) participated in stakeholder interviews. Qualitative findings indicated overall positivity in relation to accessibility, functionality, usefulness, design, and increased knowledge of advance care planning. Recommended improvements included shortened content, a comparable website for patients and families, and multilingual translations.

**Conclusions:**

The CIPP framework was effectively applied to evaluate the development and end product of an advance care planning website.Although overall findings were positive, further advance care planning website development should consider the recommendations derived from this study.

## Introduction

### Application of Electronic Health in Advance Care Planning

Integration of advance care planning (ACP) is associated with improved quality of life, better adherence to patients’ wishes, and reduced hospitalizations [1]. ACP promotes conversations about future health care needs, enacted if a person is incapable of making decisions at end-of-life that may be communicated through written documentation such as advance care directives (ACDs) [2]. Globally, technological advancements have resulted in an abundance of ACP electronic health (eHealth) apps with the emergence of ACD registries, Web-based educational material, commercial and government websites, and decisions aids [3]. Reviews have largely focused on ACP decision aids or Web-based tools for patients or community members [4,5], with demonstrated improvements in patient knowledge, preparation of treatment options, and communication of health care goals [4]. Although studies of ACP websites have shown benefits [6-9], few published studies have evaluated health professionals’ experiences and perceptions of ACP websites. Notably, studies of a computer-based ACP decision aid, accessible via a website, was helpful for patients and medical students [10-15].

### Promotion of Religious and Cultural Sensitivity and Education

Although national ACP frameworks and guidelines promote inclusion of cultural and religious beliefs in end-of-life decision making [2,16,17], core value assumptions may differ between dominant western and minority cultural worldviews in relation to autonomy, decision making, information disclosure, and control over dying [18]. Within the diverse Australian multicultural [19] and multifaith [20] population, evidence suggests cultural sensitivities and divergent views about death, dying, and end-of-life care (EoLC) among Dutch and Italian migrants [21], people of Sudanese [22], African [23], Chinese [24,25], and Indian [26,27] backgrounds, and Aboriginal and Torres Strait Islander peoples [28-32]. This has implications for health care providers. EoLC, therefore, requires a considered approach, assimilating knowledge and awareness of the importance of culture, religion, spiritual beliefs, and backgrounds [33]. Education of health professionals about cultural differences expressed in ACP, communication training [34], and knowledge acquisition skills are essential to providing culturally appropriate care [35].

### The ACP*Talk* Website

To address this gap, an Australian-based website, ACP*Talk* [36], was funded to support health professionals in conducting ACP conversations with people from diverse religious and cultural backgrounds. Goals of the website were to (1) create a Web-based resource that would provide religion-specific information for health professionals conducting ACP conversations with people from diverse religious and cultural backgrounds and (2) complement existing Web-based ACP resources by providing links to religious and cultural information readily available on the Internet. A website was deemed suitable, given the necessity to create a resource that would be freely accessible, in contrast to most ACP decision aids that are proprietary or not publically available [5]. ACP*Talk* was tailored to meet the needs of the Australian-based population, consistent with the goals of the *National Palliative Care Strategy Supporting Australians to Live Well at End of Life* [37] by addressing barriers to uptake of ACP, in this case religious and cultural facets.

### Evaluation

Systematic evaluation is required to assess the effectiveness, efficiency, and appropriateness of activities [38], including the development and implementation of new eHealth apps and programs. Key evaluation standards examine the utility, feasibility, propriety, and accuracy of programs [39,40]. Although numerous evaluation approaches exist [41], evaluations should be tailored to examine specific objectives, processes, and outcomes. Program logic models [42] are frequently used in project evaluation to assess planning, implementation, and outcomes [43-46]. However, they are often constrained by the assumption of linear relationships that are presumed within the logic model [47]. The context, input, process, and product (CIPP) model is a comprehensive framework initially developed by Stufflebeam in the 1960s that may be utilized to conduct formative and summative evaluation [48]. The framework is not inhibited by assumptions of logic models [47] and is underpinned by the principles of the Joint Committee on Standards for Educational Evaluation [40]. CIPP has been widely used to evaluate health care services [49-51], educational and training programs [47,52-54], and webinars [55].

The purpose of this paper was to present a formative and summative evaluation of an ACP website, ACP*Talk,* using the CIPP framework.

## Methods

### Context, Input, Process, and Product Framework

The CIPP framework consists of four evaluation types that are summarized below [48]:

- Context evaluation is used to judge and assess project needs, problems, assets, opportunities, and contextual conditions
- Input evaluation is concerned with program planning through assessing budgets, procedural plans, feasibility, challenges, and targets
- Process evaluation examines implementation of planned and actual processes
- Product is concerned with examining program outcomes, how the program effectively addressed needs, and achieved its goals

**Figure 1:**
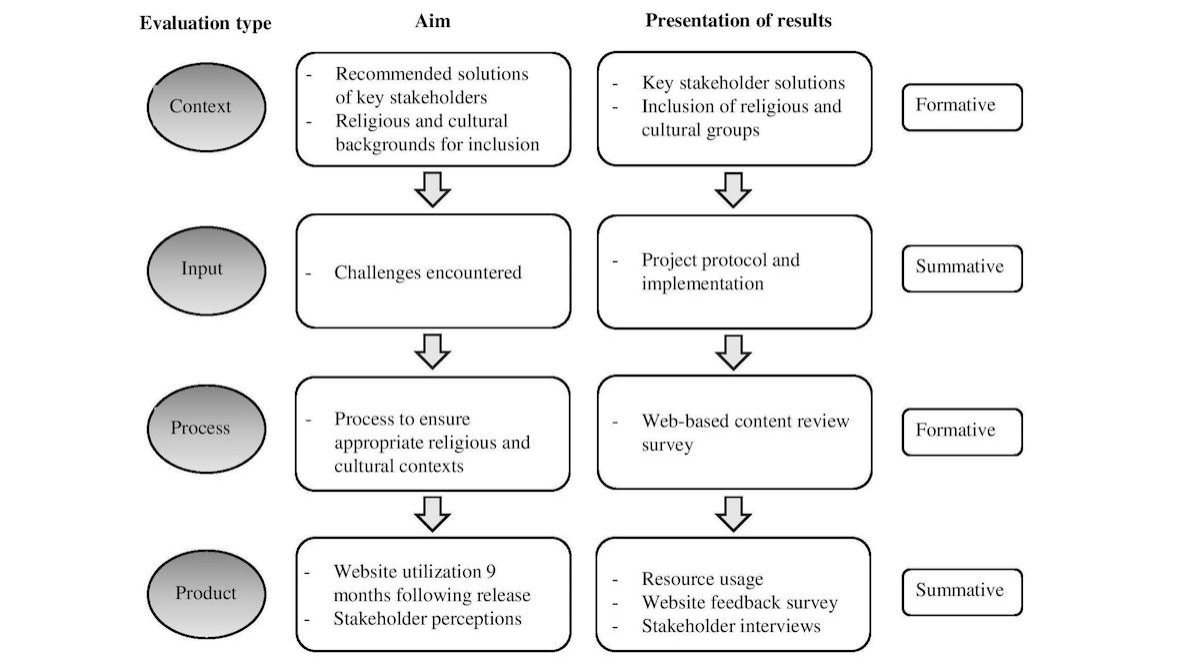
ACP*Talk* evaluation schema.

The CIPP model enables evaluators to design specific core questions for each evaluation type that are relevant to the intended project to be assessed. On the basis of the ACP*Talk* project, evaluation questions were devised (Table 1). Rationale and methods for each evaluation type are presented; mixed methods consistent with the CIPP framework were employed. Schematic flow of the ACP*Talk* evaluation and presentation of results are described in Figure 1.

### Context and Input Evaluation

#### Project Protocol and Implementation

A project protocol was developed inclusive of three main phases: (1) an exploratory study, (2) religious leader interviews that would be used to derive religion-specific website content, and (3) an evaluation. The study received ethical approval by a human research ethics committee (CHREC 02-05-10-15).

Project governance was established with a project steering committee (PSC) and project working group (PWG) convened. The PWG developed project and research aims, protocol, data collection, and management procedures and reported outcomes to the PSC. The PSC consisted of expert representatives from religious and cultural organizations, universities, palliative care, and nursing researchers and general practitioners (GPs) who were responsible for providing overall project oversight, approving protocols, procedures, recommending linkages for recruitment, and website feedback. Documentation resulting from these committees included PSC meeting minutes, the project protocol, email correspondence, and reports.

**Table 1:** Overview of context, input, process, and product (CIPP) model for ACP*Talk* (ACP: advance care planning).

Type and questions			Rationale	Evaluation methods
**Context**					
	**In developing an Australian-based ACP website for health professionals focusing on religious and cultural appropriateness**			
		What were the recommended solutions of key stakeholders (Health professionals—general practitioners and nurses)?	It was important to understand the recommendations of potential website users (ie, key stakeholders) to tailor content to the intended audience	Thematic analysis of interviews with health professionals (exploratory study)
		What religious or cultural backgrounds were included in website content?	Appropriate consideration should be undertaken to ensure that the website contains religion-specific information representative of Australian-based groups, with integration of cultural information where relevant	Review of Australian cultural diversity data, conduct of an environmental scan of Australian religious and cultural ACP resources, and documentary analysis of meeting minutes
**Input**				
	What challenges were encountered during project implementation?		Creating a website integrating information representative of multiple religious and cultural groups requires an appropriate governance structure, timelines, and procedures	Documentary analysis of project protocol and meeting minutes
**Process**				
	What process was utilized to ensure that the website content supported ACP conversations within appropriate religious and cultural contexts?		It was important to ensure accuracy of different religion-specific content as this was a key project requirement	Development of website prototype, pretesting of functionality and content review by religious and cultural leaders. Analysis of religion-specific Web-based content review survey^a^
**Product**				
	How was the website used within the initial 9 months following release?		Measurement of website utilization is important to assess impact and inform strategies for improvements	Reporting of analytic data integrated in the website
	What were stakeholders’ (website users and nurses) perceptions of the website?		Obtaining stakeholder perceptions of website benefits, weaknesses, and suggestions for improvements to inform further development	Analysis of website user feedback survey^a^ and thematic analysis of stakeholder (nurse) interviews

^a^Reported as per the *Checklist* for Reporting Results of Internet E-Surveys [56] checklist.

#### Inclusion of Religious and Cultural Groups

An environmental scan [57] was conducted to examine availability and accessibility of Web-based Australian ACP resources (websites and informational booklets) that contained religious and cultural information. This was intended to assist with identifying needs, determining religious and cultural groups for inclusion in the website, and locating quality resources to be incorporated in the website. Cultural diversity statistics [19,20] were examined to determine potential religions for inclusion in the religion-specific search function of the website.

#### Stakeholder Solutions

Given that ACP conversations can occur throughout the illness trajectory [2,58], the website was tailored to health professionals; in particular GPs and general practice nurses working in primary health care with applicability to palliative care professionals.

Key stakeholders—GPs and palliative care and general practice nurses (minimum qualification clinical nurse specialists) were invited to participate in the exploratory study. Following consent, semistructured interviews were conducted using a questionnaire based on the cultural awareness framework by Jirwe [59] that outlines four components of cultural competence: awareness of diversity among human beings, an ability to care for individuals, nonjudgmental openness for all individuals, and enhancing cultural competence as a long-term continuous process. Pretesting of the interview guide for clarity and understanding was undertaken with clinical nurse educators. Health professionals were asked about their experiences of conducting ACP with people from diverse religious and cultural backgrounds, what they perceived to be important when facilitating such conversations, difficulties they encountered, and recommendations for improving these. Recommended solutions for improving difficulties are presented in this paper. Participants completed a demographics form that collected data about age, sex, years working as a health professional, health professional type, country of birth, religion, and perceived importance of religion. Recruitment strategy included purposive sampling, where cultural population demographics [60] were reviewed to target health professionals working among diverse religious and cultural population distributions across Australia. Interviews were voice recorded and transcribed verbatim.

### Process and Product Evaluation

#### Website Content and Functionality

Findings from the environmental scan [57] and health professional exploratory study informed refinement of a semistructured questionnaire guide used to interview religious leaders. Following pretesting with a religious leader of Christian and non-Christian faith, interviews with religious and cultural leaders were voice recorded and transcribed verbatim. Religion-specific content was derived from thematic analysis of interviews with 38 religious and cultural leaders.

A prototype of the website was initially built by website developers, with functional and design specifications determined by the PWG and PSC. This enabled content review and functionality testing of a religion-specific search function embedded in the website, which allowed users to select a religion and denomination (if applicable) from a predetermined drop-down list and view content pertinent to that religion. To ensure accurate and appropriate representation of religious information, content underwent an extensive review process with religious and cultural leader interviewees, representatives from state and national religious and cultural organizations, and health care organizations in specialized end-of-life roles. Reviewers were invited to view the website prototype, in particular information about their religion (if applicable), and were then invited to participate in a closed, Web-based content review survey.

#### Web-Based Content Review and Website Feedback Surveys

Although the Web-based content review survey obtained feedback about religion-specific content, a website feedback survey was used to examine stakeholder perceptions of the end product, the ACP*Talk* website. Both surveys will be reported according to the *Checklist* for Reporting Results of Internet E-Surveys [56] checklist.

Both the Web-based content review survey and website feedback survey were based on the core program evaluation standards, utility, feasibility, propriety, and accuracy [40] and elements of the Website Evaluation Questionnaire (relevance, comprehensibility, user friendliness, structure, and layout) [61]. A 5-point Likert scale with statements was developed, and responders were asked to indicate the extent to which they agree or disagree with statements through a Web-based SurveyMonkey [62] survey. Likert questions were reviewed by the PSC and pretested among peers to ensure clarity and understanding before use.

For the Web-based content review survey, questions were about accuracy of content, appropriateness to the intended audience, detail and depth, ease of reading, completeness of information, perceptions of utility, and least and most useful section. Questions were nonrandomized, located on one page, and two out of five total questions were compulsory. Answers to the least and most useful sections and comments were not compulsory.

For the website feedback survey, to examine users’ perceptions of the website, an open, voluntary, Web-based feedback survey using SurveyMonkey [62] was available for completion accessible from the website. A campaign was conducted from March 1, 2017 to May 1, 2017, promoting the website and feedback survey via Internet-based newsletters and postal mail with a prize draw offered to encourage participation. The survey asked users to indicate what state they reside in; if they were a health professional, if so what type; how they found out about the website; which parts of the website they explored; and least and most useful section. They were asked to indicate the extent to which they agree or disagree with statements based on a 5-point Likert scale that examined their perceptions of the website design, content, information, and impact on ACP knowledge and awareness. A total of 11 nonrandomized questions, seven of them compulsory, with two questions per page were asked. Responses to questions on explored website sections, most or least useful sections, and comments were not compulsory. For both surveys, consent was implied by participation, and users were only able to respond once, as determined by cookies.

#### Resource Usage

Google analytics [63] were integrated into the website to track resource usage, users’ state and territory location, number of unique visits, total visits, new or returning visitors, bounce rate, as well as time spent on the site [64].

#### Stakeholder Interviews

General practice and palliative care nurses were invited to participate in the evaluation of ACP*Talk* through national professional association newsletters and ACP-attended training sessions. Interested nurses registered via an online form. Following consent, nurses completed an instructional website exploration guide, demographics form, and a semistructured voice-recorded interview. The instructional website exploration guide was developed to ensure they had used key elements of the website before interview feedback, which included the ACP*Talk* religion-specific search function, discussion scripts, religious and cultural resources section, and the ACP law component. The demographics form requested nurses to indicate sex; age; whether they were a general practice or palliative care nurse; years of nursing experience; country of birth; if born overseas, number of years living in Australia; and religion. Nurses were asked to indicate on a 5-point Likert scale from strongly agree to strongly disagree with the statement, “Religion is important to the way I live my life.” A semistructured interview questionnaire was developed for the interviews to examine user perceptions of benefits, weaknesses, and improvements to the website (Textbox 1).

### Evaluation Analysis

Qualitative interview data (context and product evaluation) was examined using thematic analysis [65]. This involved an iterative process of data immersion, repeatedly reading transcripts, and with similar content coded into themes, supported by qualitative data management software [66]. Documentary analysis was performed to review cultural diversity data, environmental scan, and meeting minutes (input evaluation). Quantitative data obtained from Web-based surveys (process and product evaluation) were collapsed into three Likert categories for simplicity (strongly agree or agree, neutral, or strongly disagree or disagree) and analyzed as frequency distributions using statistical software. Quantitative participant demographic data (process and product evaluation) were analyzed with descriptive statistics or frequency distributions where appropriate, using statistical software [67].

**Nurse stakeholder interview questionnaire.**

1. What are your experiences of having advance care planning (ACP) conversations with people from different religious and cultural backgrounds?
2. What do you think are some of the benefits of the website? [Ask to provide an example of how the website has been useful]
3. What are some of the weaknesses? [Ask to provide an example of any issues or concerns or difficulties]
4. How do you think ACP*Talk* can be improved?
5. How do you feel the website has contributed to your understanding of ACP in a culturally diverse context?
6. Would you recommend the resource to colleagues? If no, why not?
7. Are there any additional comments or questions you have and feel would be important for the evaluation and improvement of this resource?

## Results

### Context and Input Evaluation

#### Key Stakeholder Solutions

A total of 16 health professionals (GPs: n=7, primary health nurses: n=2, and palliative care nurses: n=7) were included in the exploratory study analysis (participation rate 26%, 17/65). One participant’s interview was inaudible and therefore not included in the analysis. Characteristics are summarized in Table 2.

All interviewed GPs and nurses had ACP experiences with people across the lifespan from different religious and cultural backgrounds; in rural, urban, community, residential, and hospital settings.

In providing a resource to support religiously and culturally appropriate ACP, health professionals stated the following solutions (N indicates nurse; GP indicates general practitioner):

##### Improved Availability of Content

Although health professionals asserted the importance of nonpresumptive individualized care in facilitating ACP, they expressed solutions for enhanced religious and cultural information provision, which encompassed:

- An understanding of varied religious beliefs and related requirements, N5 stated:

I think if you had an easy guide to—like 4 or 5 dot points under some of the more common cultural or religious beliefs that would be helpful...

- Religious or cultural information outlining communication ideas to introduce ACP and decision making with different religious groups. This would include generically safe words to use and ideas for how to explain medical and technical ACP-related terms in sensitive ways. N6 exclaimed:

It would be good to have something to support you and guide you in those conversation.

- Preparatory information for dealing with potential reactions. This could include education with role plays and discussions. GP3 stated:

...you could have an education session that involved role playing...just doing examples of various ways in which there may be problems with communication or difficulties getting over certain stumbling blocks.

- Legal information applicable to one’s work jurisdiction
- Additional support contacts, for example, interpreter details
- ACP multilingual resources for patients or families

##### Well-Developed Design and Functionality

- Online, textual, and audio-visual; N7 stated:

...a video on iPad or computer that has a person from that culture speaking about how they approached advance care planning.

- Ease of access was mentioned, with GP2 requesting:

...online resources available and easily accessible, and if you're not sure, you can just go and have a look at those resources. That would be quite handy.

- Link-in with well-known palliative care websites
- Easy to follow downloadable resources guides

#### Inclusion of Religious and Cultural Groups

The environmental scan identified seven Australian-based ACP websites and seven ACP informational booklets with cultural and religious information representative of Aboriginal and Torres Strait Islander (n=5), Sikh (n=1), and Italian communities (n=1) [57]. No comprehensive Australian-based ACP website or informational booklet supporting ACP across several cultural and religious contexts was identified. Review of Australian Bureau of Statistics census data [19,20] indicated that the majority of the population were Christian (61%), which was made up of 9 denominations (Catholic, Anglican, Uniting Church, Presbyterian, Eastern Orthodox, Baptist, Lutheran, Pentecostal, other Christian). Non-Christian faiths accounted for 7% (Buddhism, Islam, Hinduism, Judaism, other-non-Christian), with 22% of people not reporting a religion. It was determined by the PWG and PSC that religion-specific website content about ACP would need to be reflective of these faith groups to be relevant to an Australian-based population.

#### Project Protocol and Implementation

The major project challenge related to difficulties in recruiting GPs to the exploratory study. Further recruitment strategies were employed, including liaison with primary health care networks to assist in recruitment and reimbursing providers operating solely under fee-for-service.

It was determined by the PSC that religion-specific content would need to incorporate heterogeneity within religions. The strategy to minimize conflicting information was attempting to interview at least two leaders from these groups so that dichotomist views could be investigated. Despite this, there were some issues with differences in beliefs within certain religions. State and national religious and cultural organizations were called upon to review content to ensure accuracy.

### Process and Product Evaluation

#### Website Content and Functionality

**Figure 2:**
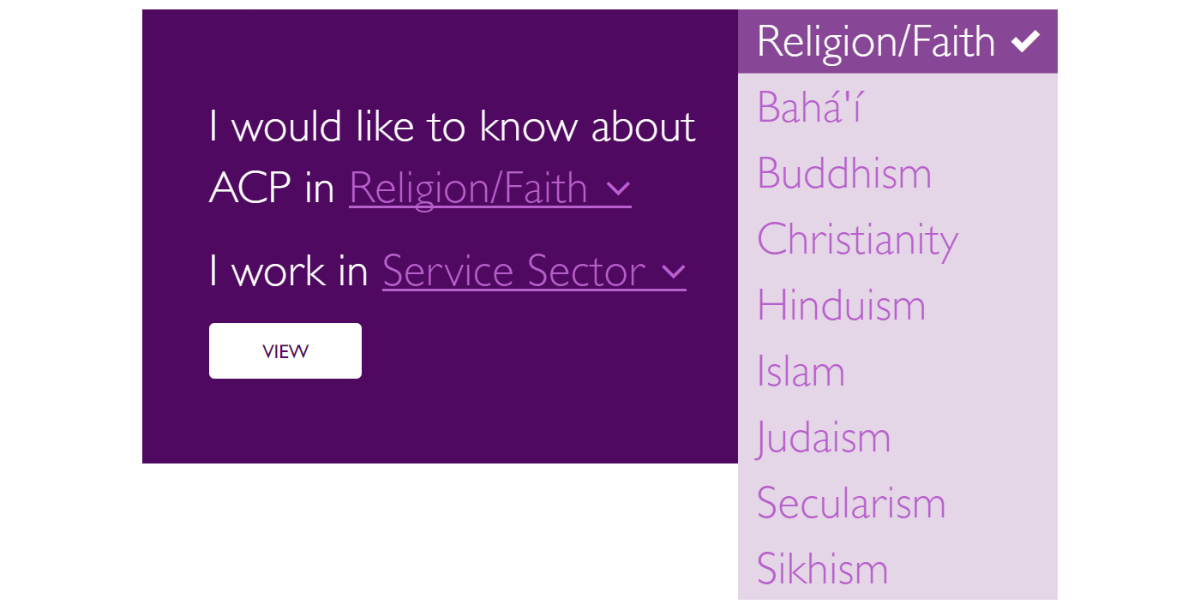
Religion-specific search function.

**Figure 3:**
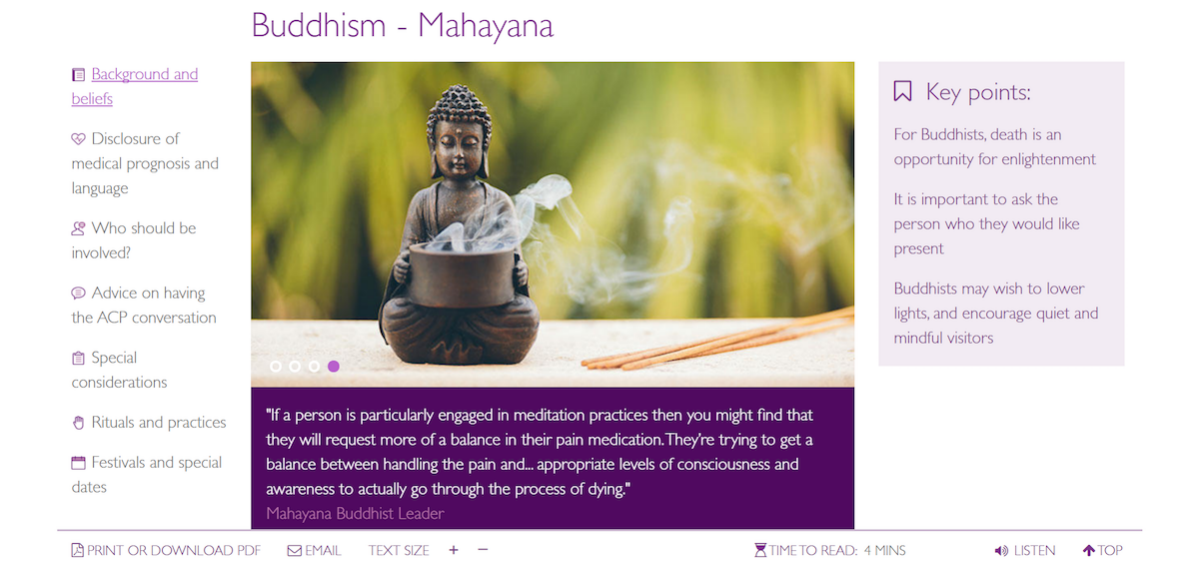
Religion-specific information.

ACP*Talk* features a religion-specific search function (Figure 2) that enables review of Christian (Anglican, Baptist, Catholic, Coptic Orthodox, Greek Orthodox, Lutheran, Presbyterian, and Uniting Church denominations) and non-Christian faiths (Bahai, Buddhism, Hinduism, Islam, Sikhism, and Secularism), reflective of population demographics [20]. Selection of religion and denomination (if applicable) functions on the website present content about background and beliefs, disclosure of medical prognosis and language, who should be involved, advice on having the ACP conversation, special considerations, rituals and practices, and festivals and dates (Figure 3).

**Table 2:** Characteristics of interviewed health professionals.

Characteristics		n (%) or median years (range)
**Type of health professional, n (%)**		
	General practitioner	7 (44)
	Primary health nurse	2 (12)
	Palliative care nurse	7 (44)
Health professional experience, median years (range)		9.5 (1.5-40)
**Sex, n (%)**		
	Male	8 (50)
Age, median years (range)		51 (38-67)
**Country of birth, n (%)**		
	Australia	11 (69)
	Overseas	5 (31)
If born overseas, time living in Australia, median years (range)		29 (9-62)
**Religion, n (%)**		
	Christian	7 (44)
	None	8 (50)
	Hindu	1 (6)
**Religion is important to me, n (%)**		
	Strongly disagree or disagree	6 (37)
	Neutral	3 (19)
	Strongly agree or agree	7 (44)

**Figure 4:**
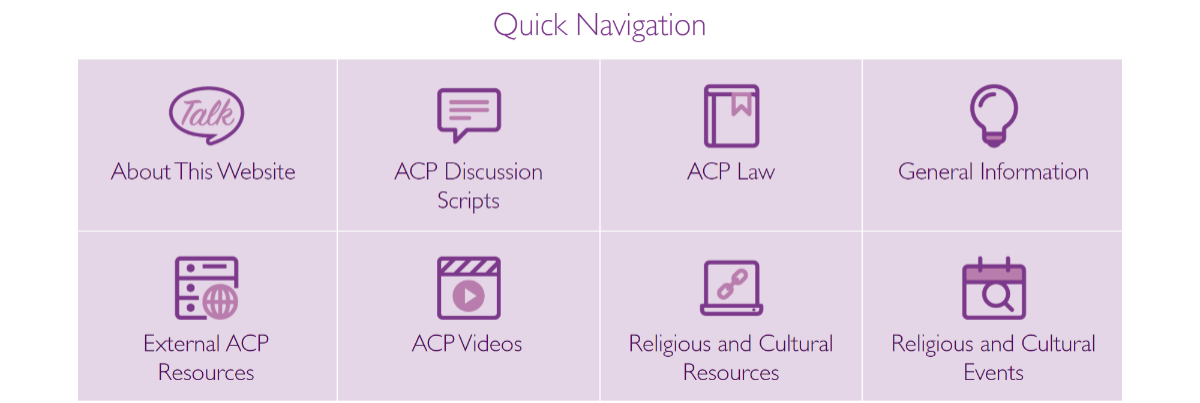
Quick Navigation.

*Quick Navigation* features the sections about this website, ACP discussion scripts that have suggestions from interviewed health professionals and religious leaders, ACP law, general information, videos, and religious and cultural events (Figure 4). To complement existing resources, an external ACP resources section features general Australian-based ACP websites, and a religious and cultural resources section features informational booklets and websites identified through the environmental scan. ACP*Talk* features a responsive design to adjust for mobile device use such as tablets and mobile phones.

#### Web-Based Content Review Survey

**Figure 5:**
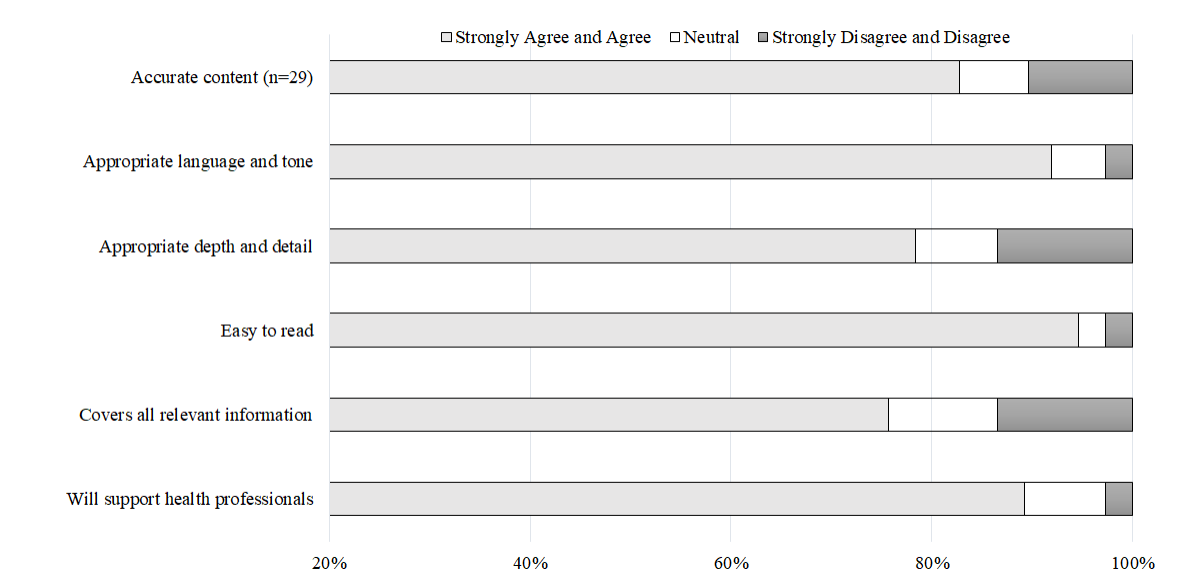
Religion-specific content reviewer responses (n=37).

A total of 37 individuals (participation rate 67%, 37/55 [survey responders/invited participants]), representative of religious and cultural organizations (n=29) and members of other interested organizations (health care n=5, community organization n=3), reviewed the website content from February 17, 2016 to April 25, 2016 and completed the Web-based survey (Figure 5). The majority of individuals strongly agree or agree that the website used appropriate language and tone (92%, 34/37), will support health professionals (89%, 33/37), and featured accurate content (83%, 24/29). Equal percentages of reviewers (24% each, 9/37) considered advice on the ACP conversation and backgrounds and beliefs as the most useful section of the religion-specific content. The least useful section indicated was special considerations (30%, 11/37). Where content reviewers felt they strongly disagree or disagree with an item and provided comment, this was addressed with amendment incorporated where relevant.

For example, interviewed religious and cultural leaders spoke about practices and rituals after death; this was not included in the initial content for inclusion in the website given that the website was focused on ACP discussions. Several reviewers of Christian and non-Christian faiths indicated it was necessary, as people may request postdeath rituals as part of the planning process; hence, this was written, and sections were reviewed. Content refinement was completed within the allocated time frame of 3 months, and no project delays occurred.

#### Resource Usage

Data from Google analytics indicated that from September 1, 2016 to May 14, 2017, there were 12,957 page views and 4260 sessions by 2920 users. Of this, 68.33%, (2911/4260) were new sessions and bounce rate, which indicates people visiting a page, and leaving without further exploration was 40.40%, (1721/4260). Most sessions were from Australia (83.45%, 3555/4260), with a bounce rate of 40.84%, 1452/3555). Most Australian users resided in the states of Victoria (46.81%, 1664/3555) and New South Wales (27.99%, 995/3555). Other countries where the website was used included the United States (5.19%, 221/4260, bounce rate 65.16%, 144/221) and Russia (3.71%, 158/4260, bounce rate 6.96%, 11/158). Users spent an average of 3.21 min on ACP*Talk.*

#### Website Feedback Survey

From March 1, 2017 to May 14, 2017, 107 Australian-based website users completed the website evaluation survey (10% participation rate [participating users/unique site visitors determined by Internet protocol address]), of which the majority (88.8%, 95/107) indicated that they were health professionals. Of those that were health professionals, approximately 57% (49/86) were nurses, 14% (12/86) allied health, 14% (12/86) GPs, 5% (4/86) medical specialists, and 10.5% (9/86) indicated other (ie, practice managers, students, and program coordinators). Table 3 shows survey responder background data.

**Figure 6:**
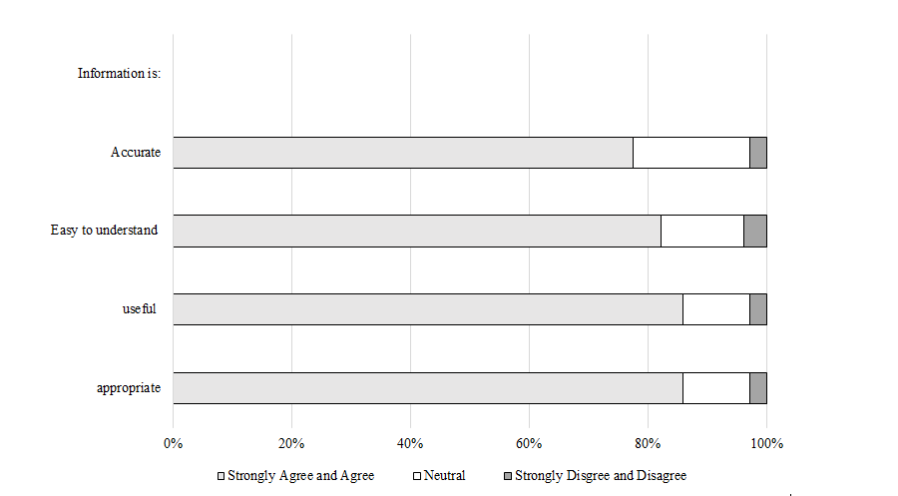
Web-based survey respondents' perceptions of website information (n=107).

**Figure 7:**
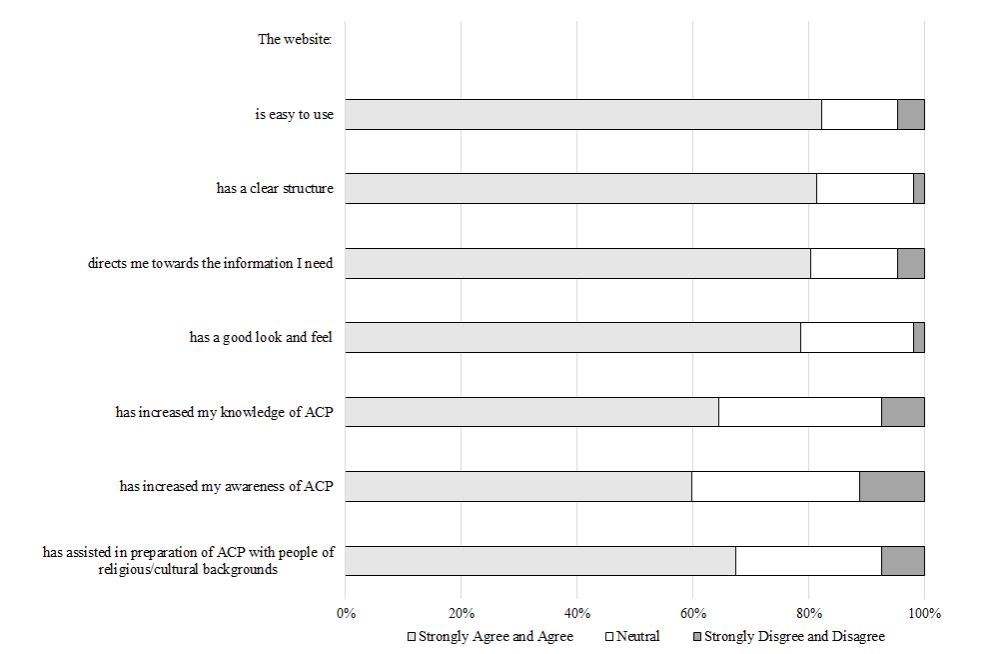
Web-based survey respondents' experiences with using the website (n=107). ACP: advance care planning.

The majority of responders resided in Victoria (n=53), found out about the website via email (n=35), and explored the home page (n=52) and religious and cultural resources (n=49). Survey responders’ perceptions of the information on the website (Figure 6) and experiences with using the website (Figure 7) are presented. The majority of survey responders viewed the website favorably. In comparison to other questions, a lower percentage, yet still the majority reported that the website increased knowledge (64.5%, 69/107) and awareness of ACP (59.8%, 64/107), with 67.3% (72/107) of responders indicating that the website assisted them in preparation of ACP with people from different religious or cultural backgrounds. Approximately 37.9% (39/103) of survey responders nominated the most useful section of religion-specific content as backgrounds and beliefs, whereas 42.2% (38/90) stated festivals and special dates as the least useful.

#### Stakeholder Perceptions

Interviews were conducted with 20 registered nurses (NS indicating nurse stakeholder; working in palliative care n=8, general practice n=10, and across both disciplines n=2). Characteristics are summarized in Table 4.

All except NS6 were experienced in offering ACP, and five nurse stakeholders (NS11, NS12, NS9, NS14, and NS26) also described ACP education roles in health care teams.

Thematic findings follow.

##### Accessibility, Functionality, and Design

Participants were complimentary about the ACP*Talk* website. Many praised its online accessibility, easy and quick navigational properties, user friendliness, clear font, and attractive format. In terms of usability, participant NS17 commented the following:

...it took me a little bit of time and I’m talking minutes rather than hours to actually work out how the website worked.

Specific appealing design features mentioned included no login required, responsive design for tablet and mobile phone use enabling access on homecare visits, (religion-specific) search function that avoided the need for scrolling, and downloadable information. Comments included the following:

For a lot of us in primary care we’re doing home visits so you’re out there with a laptop and you can flag stuff and print it off and send it out to people and that kind of stuff

NS9

I think it’s set out...clear enough that you don’t get lost within it...

NS14

**Table 3:** Web-based survey responders’ background data (ACP: advance care planning).

Survey questions		n
**State of residence**		
	New South Wales	13
	Queensland	10
	South Australia	18
	Tasmania	5
	Victoria	53
	Western Australia	8
**How users found out about the website^a^**		
	Search engine (ie, Google)	4
	Other website	7
	Email	35
	General practitioner office or staff	4
	Hospital	10
	Place of worship	1
	Electronic newsletter	7
	Printed media	22
	Professional association	14
	Other	18
**Parts of the website explored^a^**		
	Home page	52
	Religion-specific content	20
	About this website	26
	ACP discussion scripts	29
	ACP law	28
	General information	40
	External ACP resources	20
	ACP videos	16
	Religious and cultural resources	49
	Religious and cultural events	12

^a^Able to indicate more than one answer for the question.

**Table 4:** Characteristics of interviewed nurses.

Characteristics		n (%) or median years (range)
**Area of nursing, n (%)**		
	Palliative care	8 (40)
	General practice	10 (50)
	Both	2 (10)
Nursing experience, median years (range)		29 (5-44)
**Sex, n (%)**		
	Female	18 (90)
Age, median years (range)		52 (32-63)
**Country of birth, n (%)**		
	Australia	12 (60)
	Overseas	8 (40)
If born overseas, time living in Australia, median years (range)		19 (5-38)
**Religion, n (%)**		
	Christian	12 (60)
	None	6 (30)
	Mixed religions	2(10)^a^
**Religion is important to me, n (%)**		
	Strongly disagree or disagree	5 (25)
	Neutral	3 (15)
	Strongly agree or agree	12 (60)

^a^More than one religion stated by individuals.

##### Usefulness of Content

Participants commented on the comprehendible, concise, sufficient, and manageable information; and links to relevant sites. NS25 stated the following:

If I had someone culturally diverse and I could just click on that (home page) and get into the link straight away without wasting hours and searching. I could get the accurate information for whatever I needed. I really liked that.

Individuals added that it addressed a knowledge gap or was a good addition to existing cultural care resources. Especially agreeable were consistent informational headers across religions; multimedia (textual and video) and multilingual (video) learning options.

Many commended specific information provided, including different EoLC procedures associated with varied traditions, acknowledgment of two main Buddhism streams, description of Ramadan, links to state-specific legal information, interpreter contact details, religious festival dates, scripts on guiding ACP conversations, suggestions for approaching difficult situations, and advice that ACP*Talk* information is only a guideline, whereas ACP should remain individualized.

##### Improved Advance Care Planning (ACP) Knowledge, Confidence, and Cultural Awareness

Although some participants had not viewed the entire ACP*Talk* website because of time constraints or technical problems, most stated that the content examined had extended their competence and confidence in conducting or educating about ACP with people from multicultural and religious backgrounds. Comments included the following:

I think it’s assisting me in understanding the sensitivities that come with different groups and that we need to be respectful and sensitive in our conversations.

NS19

My intention is for us to use the information to frame our initial contact a little bit differently. At the moment, we tend to pretty much say the same script to everybody, which in light to this kind of information may not be the best way to go.

NS9

Many found ACP*Talk* interesting and were pleased to know of its availability if needed to increase or “refresh” ACP knowledge and competency. NS16 stated:

I would use it at the drop of a hat for any sort of unfamiliar territory.

All had already used and/or recommended ACP*Talk*, or anticipated using and recommending it to others. Although three highly ACP-experienced participants did not learn new information from ACP*Talk*, two of those welcomed using it for their ACP education. NS12 stated:

I have used it for educational purposes for myself and directed people towards it as a resource...in that way it has helped me.

Many also expected that the website would enable improved staff-client relationships through extending health professionals’ cultural ACP competency. Furthermore, occasional participants considered the website could potentially assist volunteers, patients, and families.

##### Website Challenges and Suggestions

Four participants could not think of ways to improve the website, and some offered minor design critiques, for example, on menu visuals. Some also commented on omission of materials beyond the website’s remit, for example, advance directive forms, or content which were already included, for example, an educational video on ACP with indigenous Australians. A number also described technical issues related to Web-based links or Web-enabled personal devices.

Suggested technical website improvements included increased search and home page button visibility and reduced steps (clicks) to reach Christian pages. Two participants also believed that information presented on Aboriginal and Torres Strait Islander peoples could be more prominent.

Almost all the participants thought that the website had enough information; however, individuals suggested additional information was needed on minority religions such as scientology and how to approach multicultural families before commencing ACP conversations. One who lived in an area with “huge” Chinese and Vietnamese populations queried whether information on these groups was also needed. Additionally, individuals recommended an advanced directive example, interpretations of complex legal-based ACP information, comparable websites for patients and families, and a related online chat service. Also suggested was further clarity on ACP terms through a larger glossary and acknowledging that different terms in different Australian states can refer to the same thing, as illustrated in the following quote:

Quite often when somebody is talking to you about power of attorney they’ll also talk to you about power of enduring guardianship...The medical power of attorney and some of those languages cross over.

NS9

Further recommendations included increasing content to include a “frequently asked questions” page and further condensing each religion’s information into dot points. Extending comprehension of website content to health professionals without English as a first language was also recommended through presenting more information visually and multilingual translations. NS7 stated the following:

I certainly know Doctors who are practicing in this country who speak English well enough to practice the mechanics of medicine but don’t speak English well and confidently enough to have sensitive conversations with people about advance care planning...Medical practitioners might actually benefit from...having it written in their own language.

## Discussion

### Principal Findings

This study demonstrates that the CIPP evaluation model can be applied to conduct a formative and summative evaluation of an ACP website development and end product. In terms of website development, health professionals provided the following solutions: religious or cultural information guides, communication examples, education featuring role-plays, legal information, additional support contact details (ie, interpreters), and multilingual ACP resources. Recommended design and functionality included online accessibility, audio-visuals, linkages with existing websites, and downloadable content. Most of the requested content elements and design features were integrated into the ACP*Talk* website with the exception of education featuring role-plays and multilingual ACP resources that were not within the scope of the project. The external ACP resources and religious and cultural resources sections, however, contained links to several websites that link with similar products.

Online resources identified through the environmental scan were linked to ACP*Talk*, thus meeting the project objective of complementing existing available resources and health professionals’ solutions specified in the exploratory study. Utilization of statistics from the Australian Bureau of Statistics and PSC expert opinion ensured that religion-specific content was in accordance with Australian-based faith populations, although inclusion of minority religions, that is, scientology, and further cultural information was recommended during the product evaluation.

Utilization of both state and national religious and cultural organizations ensured accuracy of religion-specific content and resolved ambiguities. Importantly, process evaluation determined that an overwhelming majority of religious leaders felt content was easy to read, used appropriate language and tone, featured accurate content, and would support health professionals in ACP. Refinements were made to content where appropriate, that is, inclusion of end-of-life rituals and practices based on feedback that this was important.

Google analytics data revealed the majority of users were new, with a bounce rate of 40%. The bounce rate is reflective of people visiting the landing page and leaving without further exploration, which may be suggestive of people either finding what they are searching for and then leaving the website or leaving the website after unintentional visitation. Australia had the highest usage, which was to be expected; interestingly Russia accounted for approximately 4% of users with a bounce rate of about 7%, indicating that residents in Russia were exploring the website.

Overall, website feedback was favorable, and the majority of survey responders were health professionals, predominantly nurses, followed by GPs and allied health and medical specialists, indicative of the target audience. The remaining survey responders may have been members of the general community, which illustrates reach of website users broader than the targeted audience and the need for such a resource within the community. Most survey responders were positive about information accuracy, ease of understanding, usefulness, appropriateness, and website design. A lower proportion of survey responders (though still a majority) felt that the website increased their knowledge and awareness of ACP and assisted in preparation of ACP with people from different religious and cultural backgrounds. This may be explained by users having existing knowledge and expertise in ACP, with experience in discussions already among diverse religious and cultural communities.

Interviewed nurse stakeholders commented on the benefits of online accessibility; user friendliness; appealing design features and functionality, including the religion-specific search function; and absence of log-in requirements. Some nurses, however, suggested that menu visuals could be improved by increasing search function and home page button visibility. Many felt that the content was useful in addressing knowledge gaps, adding to existing religious and cultural resources, and would recommend the website to others. A comprehensive list of recommendations for consideration of further Web-based development included a glossary for varying state-based legal terms, condensing religion-specific content, comparable website for patients and families, an online chat service, and multilingual translations. Though multilingual translations were mentioned in the exploratory study and end-product evaluation, this was beyond the scope of the project resources and timelines.

### Limitations

Participation rate for the exploratory health professional study was low (26%), and health professionals were not from the most culturally diverse Australian-based communities as initially intended. Although difficulties in recruitment of GPs are consistent with the literature [68,69], health professionals’ views reflected diverse involvement in ACP with people from a range of religious and cultural backgrounds. Hence, though data saturation was not reached, useful and sufficient recommendations were derived from interviews to inform website development. Due to the recruitment strategy (convenience sampling through professional newsletters and member associations), a participation rate could not be obtained for the nurse stakeholder evaluation interviews. Included nurses were reflective of palliative care and general practice disciplines, and data saturation was reached among this group with interviews ceased when no new information was emerging. Due to the interest-based nature of participation, nurse stakeholder interviews and website survey responses may be limited by responder bias. A more in-depth analysis of the website with other health professionals may have provided further recommendations.

### Comparison With Prior Work

Multiple ACP eHealth apps are available, such as governmental and commercial websites, Web-based ACD registries, and educational material [3], and published reviews have predominantly examined ACP decision aids or Web-based tools [4,5]. Broadly, evaluation of decision aids [4,5] and ACP websites [6,8-10] indicates development and acceptability of Web-based material predominantly among patients or community-based audiences. Evaluation of two community-based ACD websites revealed ACP information was increasingly sourced from the Internet; the majority of users reporting ease of use as the main reason for completing a Web-based ACD [6]. Ease of use was also reported among individuals who used *PREPARE*, a website that prepares older adults for decision making [8] and by nurses in our study. More recent evidence indicates increased ACP documentation and higher self-reported engagement in ACP by participants who used the PREPARE website [7].

In contrast to these studies, ACP*Talk* was custom-built to support health professionals in conducting ACP conversations with a particular focus on the needs of people from diverse religious and cultural backgrounds. There is limited published literature evaluating ACP websites designed for health professionals for comparison with our study. Of note, the *Making Your Wishes Known (MYWK)* computer-based decision aid is accessible via a website, developed to guide individuals through ACP with tailored education, and a tool that translates preferences and values into a medical plan for access by the treatment team [10,14]. Further studies, however, indicate usefulness of MYWK in educating medical students with reports of improved knowledge, confidence in helping patients with ACP, and greater satisfaction in learning [11,13]. Similarly, in our study, the majority of interviewed nurse stakeholders reported improved ACP knowledge, confidence, and cultural awareness, whereas most website survey responders’ felt ACP*Talk* assisted in preparation or participation of ACP with people of different religious and cultural backgrounds.

### Conclusions

This study demonstrates that all facets of the CIPP framework can be effectively applied for evaluation of eHealth technologies, specifically ACP websites, with assessment of ACP*Talk*. Results show that most users viewed the website positively in terms of design, content, and functionality and found it useful to increase knowledge and preparation for ACP with people of different religious and cultural backgrounds. Further ACP website development should consider the recommendations derived from this study, including multilingual translations and the development of comparable culturally sensitive websites tailored for patients and families, which may assist in strengthening understanding and cognizance of ACP among these populations.

## Abbreviations

ACD: advance care directive
ACP: advance care planning
CIPP: context, input, process, and product
eHealth: electronic health
EoLC: end-of-life care
GP: general practitioner
MYWK: Making Your Wishes Known
PSC: project steering committee
PWG: project working group
